# Analysis of Acrylamide in Dried Blood Spots of Lung Cancer Patients by Ultrahigh-Performance Liquid Chromatography Tandem Mass Spectrometry

**DOI:** 10.1155/2020/2015264

**Published:** 2020-05-19

**Authors:** Yahdiana Harahap, Camilla Elysia, Zenshiny Starlin, Achmad Mulawarman Jayusman

**Affiliations:** ^1^Faculty of Pharmacy, Universitas Indonesia, Depok, Indonesia; ^2^Functional Medical Staff of Pulmonology, Dharmais Cancer Hospital, Jakarta, Indonesia

## Abstract

Acrylamide (AA) is a carcinogenic substance found in food, cigarette smoke and in an environment exposed to acrylamide. This study aims to analyze AA levels in dried blood spot (DBS) samples of lung cancer patients with smoking record, without smoking record, and also in the negative blank. Analysis of AA levels was determined by liquid chromatography tandem mass spectrometry (LC-MS/MS) and DBS extraction using protein precipitation techniques. Mass detection was done using positive electron spray ionization (ESI) and multiple reaction monitoring (MRM) type with *m*/*z* values of 71.99 > 55.23 for acrylamide and *m*/*z* 260.16 > 116.04 for propranolol as the internal standard. AA levels in lung cancer patients with smoking record is in the range of 4.670 *μ*g/mL to 11.986 *μ*g/mL. AA levels in lung cancer patients without smoking record is in the range of 2.041 *μ*g/mL to 12.702 *μ*g/mL. Data on AA levels on negative blanks is in the range of 2.72 *μ*g/mL to 3.51 *μ*g/mL. The results of the independent sample *t*-test (*p* > 0.05) showed that AA levels in patients with smoking record and those without smoking record did not differ significantly. Then, the Mann-Whitney test was performed between the lung cancer group and the negative blank group and a significant difference was found between the two groups (*p* < 0.05).

## 1. Introduction

Lung cancer is one of the cancers with the biggest cause of death (1.8 million deaths, 18.4% of the total) due to a poor prognosis for this cancer [1]. About 85% of lung cancer cases are caused by smoking. Cigarette smoke contains more than 60 toxic compounds, which can cause cancerous growth [2]. One component of a carcinogen in cigarette smoke is acrylamide [3]. It has been reported that cigarette smoke contains more than 1 *μ*g of AA/cigarette [4, 5], and it is estimated that smoking can cause acrylamide intake of 3 *μ*g/kg/day [5, 6].

The International Agency for Research on Cancer (IARC) classifies acrylamide into group 2A (probably carcinogenic for humans). Formation of acrylamide is also found in foods with high carbohydrate content, such as french fries, potato chips, fried sweet potatoes, popcorn, cereals, bread, and biscuits which go through the heating stage with high temperatures above 120°C [7]. Such heating can cause a Maillard reaction between the amino acid asparagine and its reducing sugars [8, 9]. The WHO states that in the general population, the average intake of acrylamide through food is in the range of 0.3–0.8 *μ*g/kg BW/day [7].

CYP2E1 will metabolize acrylamide into glycidamide, which acts as the ultimate carcinogen. Glycidamide will bind to DNA to form DNA adducts, and this is thought to be a major cause of mutagenicity and carcinogenicity from acrylamide exposure [8, 10, 11]. Research on acrylamide continues to be conducted from animals to humans. Blood collection methods have also been developed so that the subjects can be more comfortable. Dried blood spot (DBS) biosampling method is a newly intensively developed method, and it has several advantages such as the collection process is relatively easy, only small volume of blood is required, and is minimally invasive to allow the comfort of research subjects; the analyte in the matrix that is absorbed on the DBS paper tends to be stable at room temperature so that it can be stored and distributed easily [12].

In the current study, the involved subjects are lung cancer patients with and without smoking record. This study was intended to find out whether there were differences in acrylamide levels in the two groups. Then, the group of lung cancer patients was also compared with the negative blank group. Analysis of acrylamide in DBS has never been done before. Therefore, this study conducted acrylamide analysis in DBS using LC-MS/MS with bioanalysis methods developed and validated [13].

## 2. Materials and Methods

### 2.1. Reference Standard Samples and Materials

 Acrylamide and propranolol were purchased from Sigma-Aldrich (Singapore). Formic acid, acetonitrile HPLC grade, and methanol for analysis were obtained from Merck (Darmstadt, Germany). PerkinElmer 226 paper was obtained from PerkinElmer (Waltham, USA).

### 2.2. Preparation of Solutions and Standards

Stock solutions of AA were prepared by dissolving 10 mg of AA in 10 mL ultrapure water to obtain a concentration of 1,000 *μ*g/mL. The stock solution was diluted to obtain intermediate solutions at 100 *μ*g/mL. Working solutions were prepared by dilution of the intermediate solution with ultrapure water. Working solutions were used to prepare calibration samples by diluting working solutions with whole blood to obtain a series of concentration at a range of 2.5–100 *μ*g/mL. Quality control samples of AA were prepared separately in the same procedure with the working solution at a concentration of 7.5 *μ*g/mL, 50 *μ*g/mL, and 75 *μ*g/mL for low, medium, and high quality control (QCL, QCM, and QCH). Propranolol stock solution was prepared in methanol at a concentration of 1,000 *μ*g/mL. Working solutions were prepared by dilution of the intermediate solution with methanol to obtain a concentration of 10 *μ*g/mL.

### 2.3. Sample Preparation

Blood samples were prepared by dilution of the working solution of AA to obtain concentrations in the range of 2.5–100 *μ*g/mL. Each concentration was spotted as much as 30 *μ*L on the DBS paper and then dried for 2.5 hours at room temperature. After drying, the paper was cut into two pieces and put into a tube. Then, the internal standard solution was added as much as 100 *μ*L (10 *μ*g/mL propranolol solution) and 500 *μ*L methanol. The mixture was vortexed for 1 minute and sonicated for 5 minutes. The mixture was centrifuged at 4,015 g for 1 minute, and 400 *μ*L of the supernatant was evaporated at 40°C with pressure 8 psi for 20 minutes by a vacuum equipped with nitrogen gas. The residue was reconstituted with 100 *μ*L of mobile phase 0.1% formic acid solution in water and acetonitrile (40 : 60) and sonicated for 20 minutes. The sample was vortexed for 30 seconds and centrifuged at 737 g for 3 minutes. Then, 70 *μ*L of the supernatant was inserted into a vial, and a final mixture of 10 *μ*L was injected into the LC-MS/MS system.

### 2.4. LC-MS/MS

The analysis was performed using liquid chromatography tandem mass spectrometry (LC-MS/MS) with the Acquity UPLC BEH C18 column (1.7 *μ*m, 100 mm × 21 mm). Acrylamide analysis used a mobile phase of 0.1% formic acid and acetonitrile with gradient elution. The electrospray ionization (ESI) source was operated in a positive mode with a multiple reaction monitoring (MRM) type. The MRM transition of the precursor to product ion pairs was *m*/*z* of 71.99 > 55.23 for AA and *m*/*z* 260.16 > 116.04 for propranolol as the internal standard. Capillary voltage used is 3.50 kV with 50 V cone voltage. Desolvation temperature, gas flow rate, and gas cone source flow rate were set at 400°C, 650 L/h, and 1 L/h. The inlet voltage for acrylamide and propranolol is 26 V and 35 V, respectively. The voltage in the collision chamber for each compound is 8 V and 18 V. The injection volume used is 10 *μ*L.

### 2.5. Method Validation

The validation assay was performed based on the Food and Drug Administration (2013) and the European Medicines Agency (2011) guidelines for validation of bioanalysis [14, 15].

### 2.6. Selectivity and Specificity

Selectivity and specificity were assessed by analyzing the blank whole blood and the blank whole blood spiked with AA and IS (internal standard) spotted on the DBS paper. Assessment used a minimum of six matrixes from different subjects. The peak area of the endogenous component should be less than ±20% of LLOQ's peak area and ±5% of the IS's peak area.

### 2.7. Linearity

Blank sample, zero sample, and eight concentrations of AA (2.5, 5, 7.5, 15, 37.5, 50, 75, and 100 *μ*g/mL) in whole blood for the calibration curve were prepared and spotted onto the DBS card. Each sample was analyzed by plotting the peak area ratio between the peak area of AA and IS. Each concentration was replicated three times and analyzed with the same procedure. The calibration curve is acceptable if nonzero calibrators are ±15% of the nominal concentration in each validation run except for LLOQ which is ±20% of the nominal concentration in each validation run and also 75% and a minimum of six nonzero calibrator levels should meet the criteria in each validation run.

### 2.8. Accuracy and Precision

Accuracy and precision were analyzed at four concentrations from the quality control stock (LLOQ, QCL, QCM, and QCH). Intra- and interday assessments were performed with five replicates and required a % coefficient of variance (%CV) ±15% except for LLOQ 20%, and accuracy (%diff) should be ±15% except for LLOQ ±20%.

### 2.9. Recovery

Recovery was calculated by analyzing QC samples in the whole blood and the blank whole blood sample that was spiked with analyte postextraction at three concentrations (QCL, QCM, and QCH). The peak area ratio of QC samples and those of the samples spiked with analyte postextraction was compared at each concentration to obtain the % recovery value. The steps were replicated three times for each concentration. The recovery value is acceptable if % CV is ± 15%.

### 2.10. Matrix Effect

Matrix effect was evaluated by comparing the peak area of the analyte between the working standard solution and the blank whole blood sample that was spiked with the analyte postextraction at two concentrations (QCL and QCH). The %CV of the ME should not be more than ± 15%. The standardized matrix factor values with the internal standard should obtain the acceptance range of 0.80 to 1.20.

### 2.11. Carryover

Carryover was assessed by injecting blank samples after calibration standard at the upper limit of quantification. The measured peak area should not be greater than 20% of the peak area of the analyte at lower limit of quantification (LLOQ) and 5% of the peak area of the internal standard, respectively.

### 2.12. Stability

Stock solution stability of acrylamide and propranolol was evaluated in short terms at 0, 6, and 24 hours at room temperature (25°C) and long terms at days 0, 7, 14, 21, and 28 at storage temperature (−20°C). The test was performed in three replicates, and the %diff value should not be more than 10%. Sample stability was tested by analyzing the QCL and QCH after short-term storage (kept at room temperature for 0, 6, and 24 hours) and long-term storage (at freezer (−20°C) for days 1, 10, and 15). Autosampler stability was also tested by analyzing the QCL and QCH (kept at autosampler temperature for 0 and 24 hours). The test was performed in three replicates, and the %diff and %CV value should not be more than 15%.

### 2.13. Application of the Method

This study has obtained the ethical review from the Health Research Ethics Commission of Dharmais Cancer Hospital with number 030/KEPK/III/2019. Dried blood spots were collected from 18 patients consisting of 6 lung cancer patients with a smoking record and 12 lung cancer patients without a smoking record. Blood sampling was done through a finger prick with a sterile lancet needle. The blood that has been taken was then collected in a 0.5 mL K_3_EDTA vacutainer. After that, 30 *μ*L blood was immediately spotted on the DBS paper and dried for about 2.5 hours at room temperature. The dried DBS sample was stored in a sealed bag containing a desiccant until the time for analysis.

Data processing was performed on the results of acrylamide analysis in DBS samples of lung cancer patients with and without smoking record. Acrylamide levels were calculated using the linear regression equation that has been made. Then, a statistic test was performed using the independent sample *t*-test and the Mann–Whitney test to see any significant differences between groups.

## 3. Results and Discussion

### 3.1. Chromatography System and Sample Preparation

**Figure 1:**
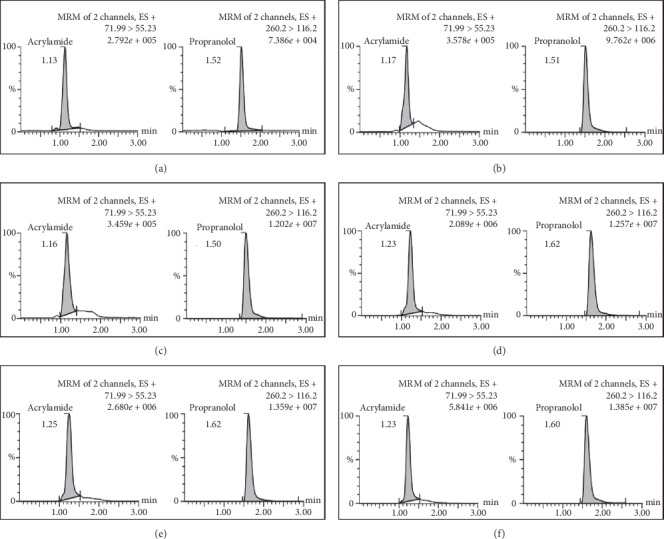
Chromatogram of (a) blank, (b) zero, (c) LLOQ, (d) QCL, (e) QCM, and (f) QCH.

**Figure 2:**
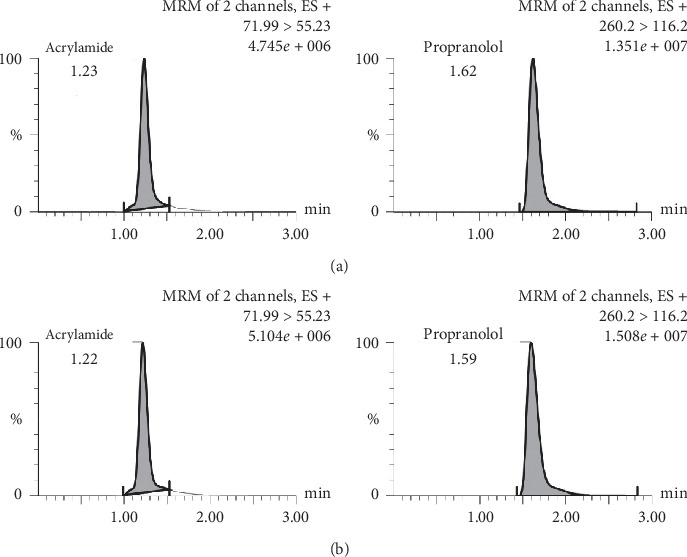
Chromatogram of the analyte in the sample: (a) sample of subject SK05 and (b) sample of subject SK07.

In this study, LC-MS/MS is used with a triple quadrupole type mass analyzer and multiple reaction monitoring (MRM) mass spectrometry mode. MRM is used because it is very well used in analysis on a complex matrix such as DBS. The mobile phase used was 0.1% formic acid and acetonitrile with a gradient elution profile as shown in Table 1. Elution was performed using a gradient-type elution with a total analysis time of 3 minutes. The chromatograms of blank, zero, LLOQ, QCL, QCM, and QCH are shown in Figure 1. The chromatogram of the analyte in the sample is shown in Figure 2.

Preparation method is a very important method in analysis, especially in DBS samples which contain many impurities from whole blood components in it that can interfere the analysis of the compound. Therefore, suitable preparation methods are needed so that they can separate the analyte from the matrix. The volume of blood spotted on the DBS paper and each step of the extraction method were also optimized, and a sample preparation method was obtained as described above. Precipitation protein techniques (PPT) have advantages over other sample preparation methods such as SPE and LLE, that is, rapid, simple, and may be adaptable to high-throughput screening.

### 3.2. Method Validation

Full validation assay has been conducted in the previous study and in the same laboratory. The linearity of the calibration curve was determined by plotting the peak area ratio (*y*) of the analyte to the internal standard versus the nominal concentration (*x*) of AA. The calibration curves were linear over the concentration range of 2.5–100.0 *μ*g/mL for AA. As shown in Table 2, the intra- and interassay precision and accuracy experiments performed on LLOQ and QC samples (QCL, QCM, and QCH) fulfilled the requirement of the EMA and FDA, the average %diff and %bias requirements not to exceed ± 15% unless the average LLOQ %diff concentration does not exceed ± 20%. Within run and between run imprecisions were in the 1.89 to 7.07% and 4.12 to 9.51% ranges, respectively. Accuracy within run and between run was estimated at a %bias 6.12 to 10.35% and 9.88 to 11.73%. LC-MS/MS methods should exclude any matrix effects. The effect of the matrix was shown by analysis between the analyte area added after the blank extraction process compared to the area of the standard solution in the solvent. This method showed an acceptable matrix effect ranging from 96.33 to 105.68% with variability (CV) under 10%. The recovery was obtained in at percentages of 95.51%, 84.61%, and 90.61% with the %CV 0.68%, 1.56%, and 0.68%. Data of recovery are shown in Table 3.

### 3.3. Analysis of Acrylamide in DBS Sample

**Figure 3:**
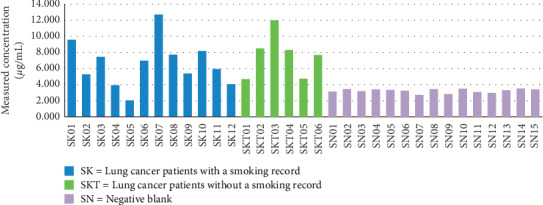
Graph of interpatient acrylamide levels and negative blank in DBS samples.

All samples were analyzed using the validated method as described above, and the concentration of AA was calculated. Graph results of the AA level in lung cancer patients without a smoking record, lung cancer patients with smoking record, and negative blank are shown in Figure 3. Overall, the lowest and the highest acrylamide levels were 2.042 *μ*g/mL and 12.70 *μ*g/mL. Lung cancer patients with smoking record have an average of acrylamide levels of 7.64 *μ*g/mL and without smoking record have an average of acrylamide levels of 6.6 *μ*g/mL. Independent sample *t*-test was performed, and the result showed that AA levels in patients with and without smoking record did not differ significantly. This insignificant difference is due to several things, including lung cancer patients with smoking record have stopped smoking with various time periods. Other factors are sources of AA in addition to smoking that can increase AA levels, such as lifestyle (diet) and cigarette smoke environment. Exposure to AA can be from foods containing AA, cigarette smoke, even drinking water, and all of that is a daily consumption of the people. Data on food consumption habits of lung cancer patients are shown in Table 4. DBS of SK07 contained acrylamide in the highest concentration of 12.07 *μ*g/mL. Based on data obtained from the questionnaire, SK07 was the patient who consumed acrylamide-containing foods the most, moreover by becoming passive smoker for years in the family and workplace environment. DBS of SK05 had AA in the lowest concentration of 2.042 *μ*g/mL. This was in accordance with the data obtained from questionnaire that SK05 was the patient who consumed acrylamide-containing foods the least and was a nonpassive smoker.

**Figure 4:**
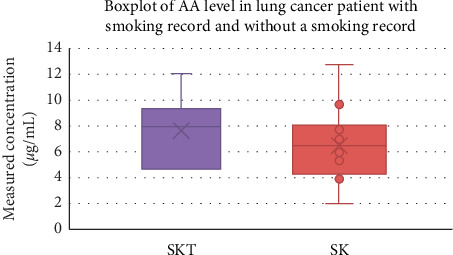
Graph results of AA level in lung cancer patients with smoking record and without a smoking record; SKT is a lung cancer patient with smoking record, and SK is a lung cancer patient without smoking record.

AA being absorbed by the body will be metabolized by CYP2E1 into its metabolites, so that the metabolic rate of AA is strongly influenced by the activity of this enzyme. Based on research conducted by Pelle [16], high levels of AA in lung cancer patients without smoking record can also be associated with polymorphism, T-allele rs2480258 which plays a role in reducing the functional activity of CYP2E1 which can affect the rate of AA metabolism in the human body. Graph results of AA level in lung cancer patients without a smoking record and lung cancer patients with smoking record are shown in Figure 4.

**Figure 5:**
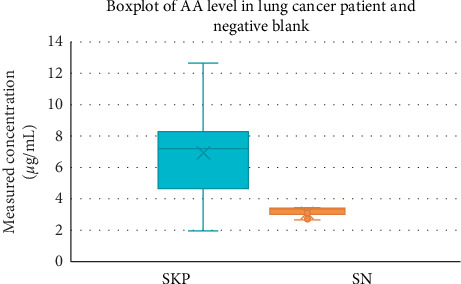
Graph of the analysis of acrylamide levels between the lung cancer patient group and the negative blank; SKP is a lung cancer patient, and SN is the negative blank.

Mann–Whitney test was then performed on acrylamide levels in lung cancer patients compared with negative blanks that did not have cancer disease and had minimal acrylamide food consumption. Results of the analysis (*p* < 0.05) showed a significant difference between acrylamide levels in lung cancer patients and their negative blanks. The graph of the analysis of acrylamide levels between the lung cancer patient group and the negative blank group can be seen in Figure 5.

## 4. Conclusion

This analytical method can be used to analyze acrylamide and propranolol as a standard in DBS samples using LC-MS/MS with LLOQ of 2.5 *μ*g/mL. AA levels in lung cancer patients ranged from 2.041 *μ*g/mL to 12.702 *μ*g/mL. Independent sample *t*-test results (*p* > 0.05) showed that acrylamide levels in lung cancer patients with and without smoking record did not differ significantly. Mann–Whitney test results (*p* < 0.05) showed a significant difference between acrylamide levels in lung cancer patients and their negative blanks.

## Figures and Tables

**Table 1:** Profile elution of the mobile phase.

Minute	Flow rate (mL/minute)	Mobile phase A (%)	Mobile phase B (%)	Minute
0.00	0.2	40	60	0.00
0.05	0.2	60	40	0.05
1.00	0.2	60	40	1.00
1.20	0.2	50	50	1.20

**Table 2:** Summary of the full validation result.

Analyte	QC	Conc. (*μ*g/mL)	Precision (%CV)		Accuracy (%bias)		Matrix effect		Internal standard-normalized matrix factor	
			Within run	Between run	Within run	Between run	Mean % ± SD	%CV	Mean % ± SD	%CV
AA	LLOQ	2.50	7.07	9.51	−10.35	−11.73				
	QCL	7.50	3.89	4.12	6.12	9.88	105.68 ± 12.34	11.68	0.91 ± 0.13	14.11
	QCM	50.0	3.12	4.48	9.49	13.71				
	QCH	75.00	1.89	5.41	−6.29	11.75	96.33 ± 8.77	9.11	0.98 ± 0.10	10.11

Propranolol							117.05 ± 5.77	4.93		

QC: quality control, LLOQ: lower limit of quantification, QCL: low level, QCM: medium level, QCH: high level, and CV: variability, number of experiments.

**Table 3:** Data of acrylamide recovery process.

Exact concentration (ng/mL)	Area		% absolute recovery	Average (%)	SD	CV (%)
	Without extraction	With extraction				
**7500.00**	54671.004	52228.059	95.53	95.51	0.65	0.68
	55565.730	53426.156	96.15			
	56385.820	53478.938	94.84			

**50000.00**	315153.000	271452.594	86.13	84.61	1.32	1.56
	324646.469	272028.719	83.79			
	333239.719	279632.250	83.91			

**75000.00**	382069.188	346540.781	90.70	90.61	0.62	0.68
	368825.469	331795.656	89.96			
	372055.938	339249.281	91.18			

**Table 4:** Data on food consumption habits of lung cancer patients.

Subject code	Food consumption					Total
	Fried products	Bread products	Coffee	Popcorn	Fast food	
SK01	1	4	0	0	0	5
SK02	1	4	0	0	0	5
SK03	0	3	0	0	0	3
SK04	1	2	0	0	2	5
SK05	1	2	0	0	0	3
SK06	2	3	3	0	1	8
SK07	0	4	4	1	1	10
SK08	2	1	0	0	0	3
SK09	3	1	0	0	0	4
SK10	0	1	0	0	0	1
SK11	2	3	1	0	2	8
SK12	1	3	2	0	0	6
SKT01	1	2	0	0	1	4
SKT02	1	4	0	0	0	5
SKT03	1	2	0	0	0	3
SKT04	2	1	0	0	1	4
SKT05	1	4	3	0	0	8
SKT06	1	1	0	0	1	3
					Average	5

## Data Availability

The data used to support the findings of this study are included within the article.
